# The SET Domain Protein, Set3p, Promotes the Reliable Execution of Cytokinesis in *Schizosaccharomyces pombe*

**DOI:** 10.1371/journal.pone.0031224

**Published:** 2012-02-08

**Authors:** Stefan Rentas, Reza Saberianfar, Charnpal Grewal, Rachelle Kanippayoor, Mithilesh Mishra, Dannel McCollum, Jim Karagiannis

**Affiliations:** 1 Department of Biology, University of Western Ontario, London, Ontario, Canada; 2 Temasek Life Sciences Laboratory, The National University of Singapore, Singapore, Singapore; 3 Department of Molecular Genetics and Microbiology, University of Massachusetts Medical School, Worcester, Massachusetts, United States of America; University of Cambridge, United Kingdom

## Abstract

In response to perturbation of the cell division machinery fission yeast cells activate regulatory networks that ensure the faithful completion of cytokinesis. For instance, when cells are treated with drugs that impede constriction of the actomyosin ring (low doses of Latrunculin A, for example) these networks ensure that cytokinesis is complete before progression into the subsequent mitosis. Here, we identify three previously uncharacterized genes, *hif2*, *set3*, and *snt1*, whose deletion results in hyper-sensitivity to LatA treatment and in increased rates of cytokinesis failure. Interestingly, these genes are orthologous to *TBL1X*, *MLL5*, and *NCOR2*, human genes that encode components of a histone deacetylase complex with a known role in cytokinesis. Through co-immunoprecipitation experiments, localization studies, and phenotypic analysis of gene deletion mutants, we provide evidence for an orthologous complex in fission yeast. Furthermore, in light of the putative role of the complex in chromatin modification, together with our results demonstrating an increase in Set3p levels upon Latrunculin A treatment, global gene expression profiles were generated. While this analysis demonstrated that the expression of cytokinesis genes was not significantly affected in *set3Δ* backgrounds, it did reveal defects in the ability of the mutant to regulate genes with roles in the cellular response to stress. Taken together, these findings support the existence of a conserved, multi-protein complex with a role in promoting the successful completion of cytokinesis.

## Introduction

The successful completion of cytokinesis requires the intricate interplay of gene products ranging from signaling molecules to elements of the cytoskeleton. Taken together these regulatory mechanisms ensure that cytokinesis occurs in a faithful and dependable manner once every cell cycle and over wide-ranging growth conditions. In *Schizosaccharomyces pombe*, just as in more developmentally complex eukaryotes, cytokinesis is critically dependent on the assembly and constriction of a contractile actomyosin ring [1], [2]. The assembly of the ring occurs at the onset of mitosis through the formation of cortical nodes comprised of proteins with roles in nucleating and assembling actin filaments [3]. Once assembled into a contractile ring, the constriction of the filaments is controlled through the action of a regulatory module referred to as the septation initiation network (SIN). The SIN is comprised of a GTPase-regulated signaling cascade that is involved in proper ring assembly, temporal co-ordination of ring constriction, as well as the deposition of the division septum [4], [5].

Given the importance of cytokinesis in cellular growth and development, it is not surprising that evidence supporting the existence of a cytokinesis monitoring system has emerged in *S. pombe*. This monitoring system has the capacity to 1) halt cell cycle progression at G2/M transition, and 2) stabilize the contractile actomyosin ring upon perturbation of the cell division machinery [6], [7], [8], [9], [10], [11]. Together, these mechanisms ensure that cytokinesis is complete before a new cell cycle begins. Important regulators of the cytokinesis monitoring system include the SIN itself, the Cdc14 family phosphatase, Clp1p, the 14-3-3 protein, Rad24p, and the kinase, Lsk1p [8], [9], [10], [12], [13], [14].

The critical role of these components can be observed experimentally through the treatment of fission yeast cells with low doses of the actin depolymerising drug, Latrunculin A (LatA) [8], [10], [12], [13], [14], [15]. At the concentrations used (20–50 times less than that needed to completely depolymerize the actin cytoskeleton) such treatment leads to a Clp1p and Rad24p dependent delay in mitotic entry and the extended activation of the SIN. This leads to a prolonged cytokinesis-competent state that is characterized by continuous repair and re-establishment of the actomyosin ring [8], [10], [11], [13]. Abrogation of this system in *clp1Δ* or *rad24Δ* mutant backgrounds results in cytokinesis failure and the subsequent generation of inviable, multi-nucleate cells [8], [10].

In order to identify other genes with roles in promoting the reliable execution of cytokinesis, a library of *S. pombe* gene deletion mutants was screened for hyper-sensitivity to LatA (M. Mishra and D. McCollum, unpublished). One of the identified genes – defined by the annotated *S. pombe* ORF, SPCC1235.09 – encoded a WD repeat protein orthologous to human TBL1X, which is known to form part of a histone-deacetylase complex with both the MLL5 and NCOR2 proteins [16]. Since knockdown of *TBL1X*, *MLL5*, or *NCOR2* results in cytokinesis failure in HeLA cells [16] we were interested to determine if an orthologous complex might also exist in fission yeast.

In this report we present the results of the molecular and genetic analysis of the fission yeast orthologues of *TBL1X*, *MLL5*, and *NCOR2*, and show that – just like their human counterparts – they are indeed important for the reliable execution of cytokinesis. Furthermore, in light of the predicted role of the complex in chromatin modification, we performed global gene expression profiling. While this analysis demonstrated that cytokinesis genes were not significantly affected, it did reveal that the *set3Δ* mutant was impaired in its ability to modulate the expression of stress response genes. The relevance of these findings within the context of understanding the relationship between cytokinesis failure, aneuploidy, and cancer progression is discussed.

## Results

### The fission yeast Hif2p, Set3p, and Snt1p proteins share sequence similarity with the human TBL1X, MLL5, and NCOR2 proteins, respectively

To identify novel regulators of cytokinesis, a library of fission yeast gene deletion mutants was screened for hypersensitivity to LatA (M. Mishra and D. McCollum, unpublished). LatA treatment results in the depolymerization of actin filaments through the sequestration of actin monomers [15]. At low concentration (0.2–0.5 µM, or approximately 20–50 times less than that needed to completely depolymerize the actin cytoskeleton) LatA can be used to impede actomyosin ring constriction and activate the cytokinesis monitoring system. For these reasons LatA treatment can be used as a tool in genetic screens to identify mutants defective in cytokinesis [8], [10], [12], [13].

**Figure 1:**
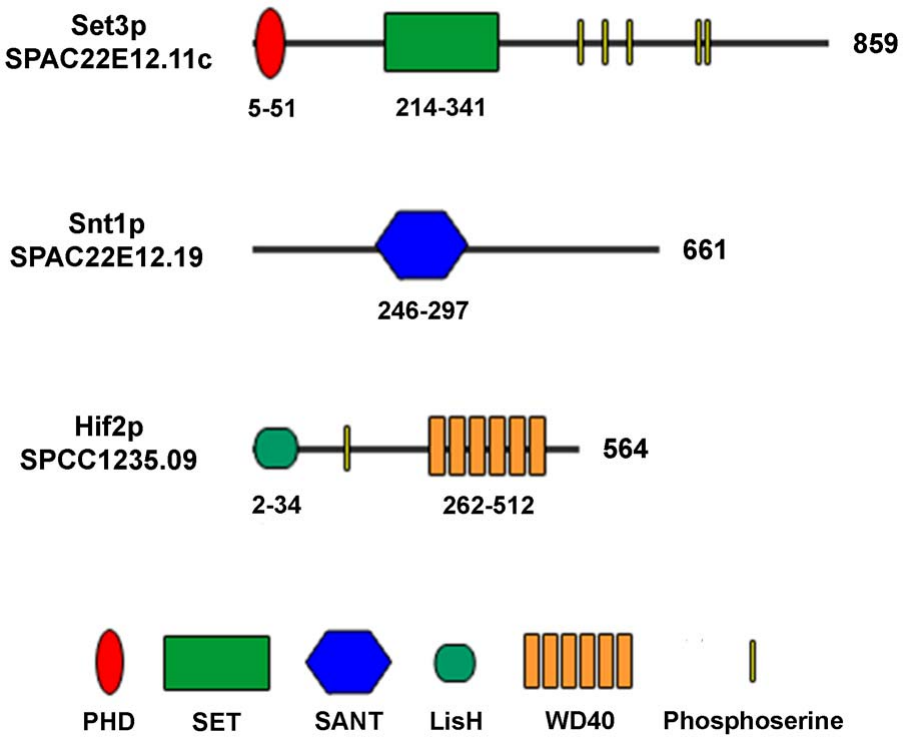
Domain structure of Set3p, Snt1p, and Hif2p. Structures are based upon Uniprot database predictions. Arabic numerals to the right of each schematic indicate the length of the protein in amino acids. Arabic numerals below each schematic indicate the amino acid position of that domain within the protein. Schematics are not drawn to scale.

This reverse genetic approach identified a strain bearing a deletion in the annotated open reading frame, SPCC1235.09, which encodes a WD repeat domain protein (Figure 1). Reciprocal BLAST searches revealed that the encoded gene product was the likely orthologue of human TBL1X (19% identity and 58% similarity; Figure S1). Interestingly, TBL1X exists in a histone deacetylase complex together with the MLL5 and NCOR2 proteins [16]. Even more intriguing was the fact that knockdown of either the *TBL1X*, *NCOR2*, or *MLL5* genes in HeLa cells results in increased rates of cytokinesis failure [16]. Furthermore, the budding yeast orthologue of the WD repeat protein also exists in a well characterized histone deacetylase complex [17]. This suggested the existence of an evolutionarily conserved multi-protein complex with a role in the faithful and reliable execution of cytokinesis.

To identify potential *S. pombe* orthologues of MLL5 and NCOR2, reciprocal BLAST searches were performed. This analysis identified two open reading frames, SPAC22E12.11c and SPAC22E12.19, which encode proteins with significant similarity to human MLL5 and NCOR2, respectively (Figure 1; Figure S1). We have named the SPCC1235.09, SPAC22E12.11c and SPAC22E12.19 open reading frames, *hif2*, *set3* (*set* domain containing), and *snt1* (*s*a*nt* domain containing), respectively.

A representation of the fission yeast Hif2p, Set3p, and Snt1p proteins and their conserved domains are shown in Figure 1. These domains typically function in chromatin remodeling. For instance, the SET domain infers possible histone methyltransferase (HMT) activity while the plant homeodomain zinc finger (PHD) is thought to assist in protein interaction with nucleosomes [18], [19]. The SANT domain is a motif found in numerous co-repressor proteins and has been proposed to recognize unmodified histone tails [20]. Lastly, the LisH domain has been shown to be important in binding to hypoacetylated histone H4 tails [21].

### *hif2Δ*, *set3Δ* and *snt1Δ* mutants display cytokinesis defects upon perturbation of the cell division machinery

**Figure 2:**
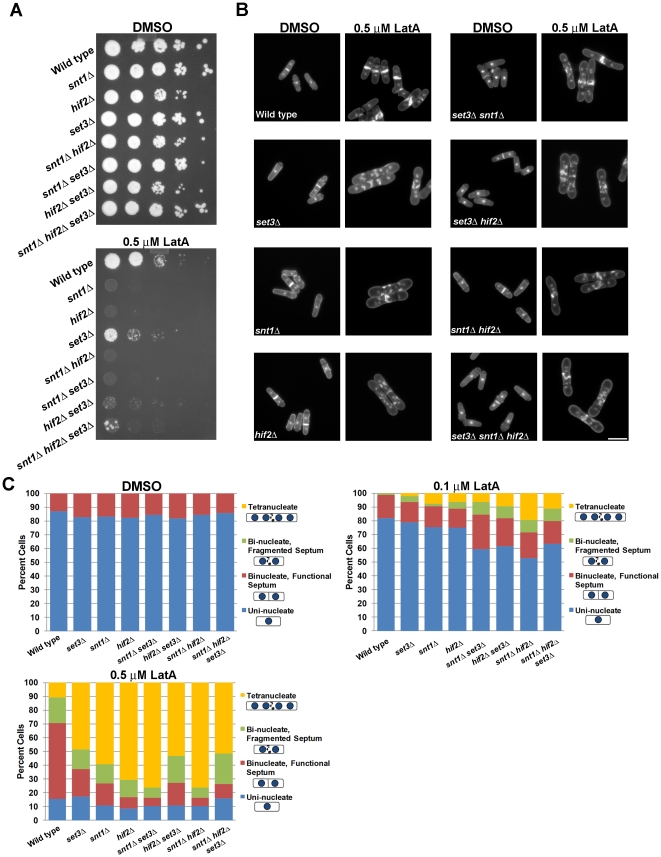
*hif2*, *set3*, and *snt1* deletion mutants are hyper-sensitive to LatA treatment. (**A**) Ten-fold serial dilutions of logarithmically growing cells of the indicated genotype were plated onto YES plates containing 0.5 µM LatA or DMSO (solvent control) at 30°C for 3 d. (**B**) Cells of the indicated genotype were grown to mid-log phase at 30°C and then treated with 0.5 µM LatA for 5 h before being fixed and stained with DAPI (nuclei) and aniline blue (cell wall/septa). Bar, 10 µm. (**C**) Quantitation of phenotypes of cells treated as in B. Between 200 and 500 cells were counted for each genotypic class.

If Hif2p, Set3p, and Snt1p act as part of a protein complex with a common function, then one would expect the respective loss of function mutants to exhibit similar phenotypes. To determine if this were the case, *hif2Δ*, *set3Δ*, and *snt1Δ* strains, as well as the respective double and triple mutant strains, were grown to mid-log phase in liquid YES at 30°C and 10-fold serial dilutions were plated onto YES plates containing LatA or DMSO (solvent control). Interestingly, all mutants displayed a reduced capacity for growth in the presence of LatA in comparison to wild type (Figure 2A).

Next, the cells were examined at the microscopic level to determine if the observed sensitivity to LatA was related to defects in cytokinesis. The deletion mutants were treated with LatA (0.5 µM) or DMSO (solvent control) for 5 hours in liquid YES growth medium, and subsequently fixed and stained with DAPI and aniline blue to visualize the nucleus and cell wall/septum, respectively [22]. The cells were classified into four different phenotypic categories: i) uni-nucleate cells, ii) bi-nucleate cells with a morphologically normal septum (i.e. the septum completely bisects the cell), iii) bi-nucleate cells with a fragmented septum (i.e. the septum is non-functional and does not completely bisect the cell), and iv) tetra-nucleate cells.

As shown in Figure 2B *hif2Δ*, *snt1Δ*, *set3Δ* and wild-type strains did not display any obvious growth defects when cultured in the presence of DMSO. In contrast, large proportions of *hif2Δ*, *snt1Δ*, and *set3Δ* mutant cells (49–75%) displayed a tetra-nucleate phenotype when treated with 0.5 µM LatA, whereas only 11% of wild type were tetra-nucleate (Figure 2B,C). Importantly, in support of the hypothesis that the three gene-products work within the same pathway, no significant synthetic effects were observed when the respective double and triple gene deletion mutants were assayed using only 0.1 µM LatA (Figure 2C). Moreover, genetic analysis with strains bearing mutations in genes encoding various cytokinesis regulators (*myo2-E1*, *cdc12-112*, *cdc15-140*) revealed specific synthetic interactions between the *set3*, *snt1*, and *hif2* gene deletions and the temperature sensitive *cdc15-140* mutation. The presence of either the *hif2Δ*, *snt1Δ*, or *set3Δ* gene deletions reduced the restrictive temperature of *cdc15-140* strains to a similar extent (but did not affect the restrictive temperatures of *myo2-E1* or *cdc12-112* strains) (Figure S2). Taken together, the similarities in phenotype observed in *hif2Δ*, *snt1Δ*, and *set3Δ* gene deletion mutants are consistent with a model in which the three proteins modulate a common biological process that influences the successful completion of cytokinesis in *S. pombe*.

### Set3p works in parallel to other regulators of the cytokinesis monitoring system

**Figure 3:**
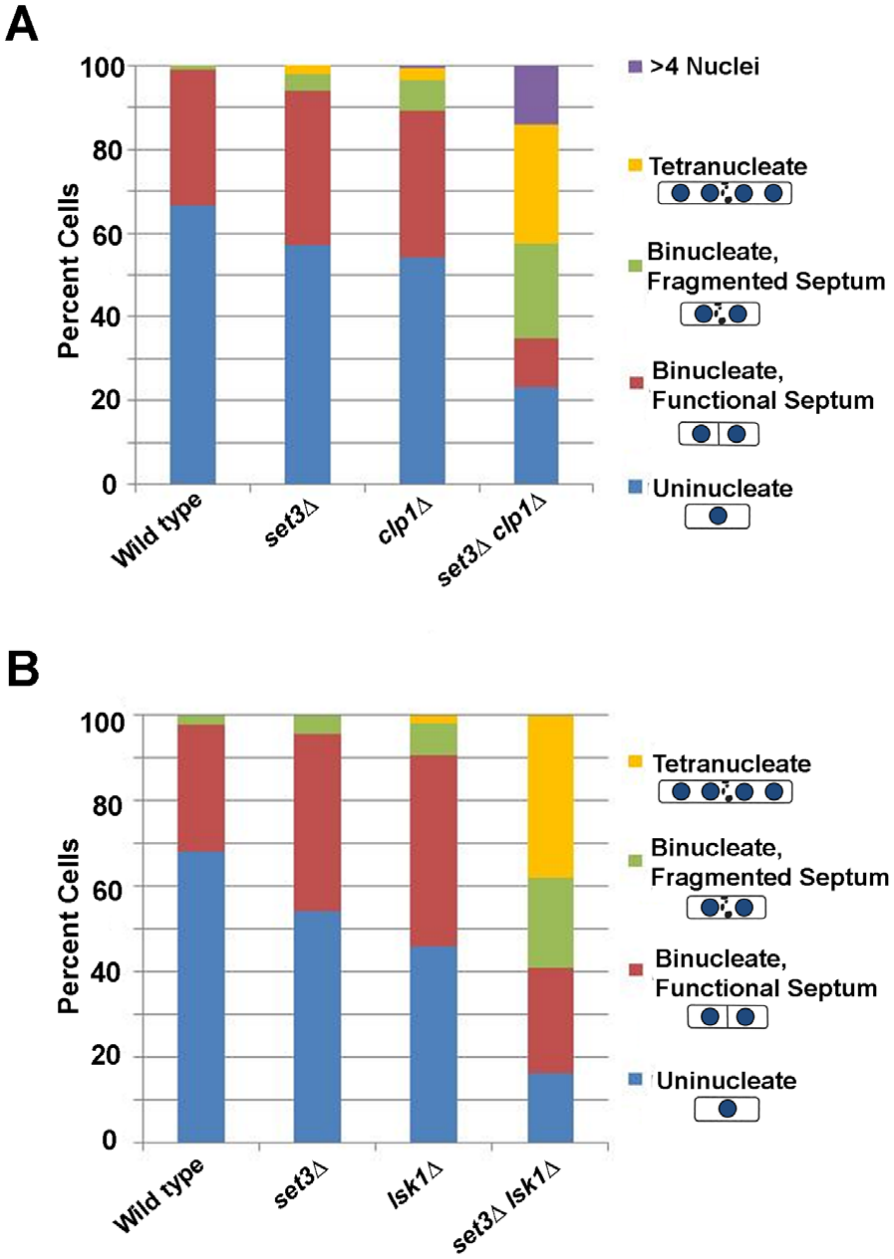
*set3* functions in a pathway parallel to those defined by *clp1* and *lsk1*. Cells of the indicated genotype were grown to mid-log phase at 30°C and then treated with 0.1 µM LatA for 5 h before being fixed and stained with DAPI (nuclei) and aniline blue (cell wall/septa). Two hundred cells were counted for each genotypic class.

The cytokinesis failure observed in *hif2Δ*, *snt1Δ*, and *set3Δ* mutants was reminiscent of that seen in the previously characterized cytokinesis regulators, *clp1Δ* and *lsk1Δ* [8], [13]. It was thus of interest to determine if these proteins functioned within the same pathway or in parallel to Clp1p (a Cdc14 family phosphatase) or Lsk1p (a Ser-2 CTD kinase), respectively. If Set3p functioned in a linear pathway with Clp1p or Lsk1p, one would expect *set3Δ clp1Δ* and *set3Δ lsk1Δ* double mutants to exhibit similar hyper-sensitivity to LatA relative to the respective single mutants. Conversely, if Set3p functioned in parallel, one would expect *set3Δ clp1Δ* and *set3Δ lsk1Δ* double mutants to exhibit a more severe sensitivity to LatA than either of the respective single mutants. To differentiate between these two possibilities, a wild-type strain, as well as the respective single and double deletion mutants, were treated with LatA (0.1 µM) or DMSO for 5 hours in liquid YES growth medium. This was followed by fixation and staining with DAPI and aniline blue to visualize the nucleus and cell/wall septum, respectively. Interestingly, both *set3Δ clp1Δ* and *set3Δ lsk1Δ* double mutants showed a significant increase in sensitivity to LatA (Figure 3). At a concentration of 0.1 µM both double mutants displayed a large proportion of tetra-nucleate cells with fragmented septa. In contrast, the phenotypes of single mutants at this dose were far less severe. These results are consistent with a model in which Set3p functions in parallel to both Clp1p and Lsk1p. Thus, *set3* defines a novel regulatory pathway governing the successful completion of cytokinesis in fission yeast.

### Set3p, Hif2p and Snt1p form a nuclear-localized complex

**Figure 4:**
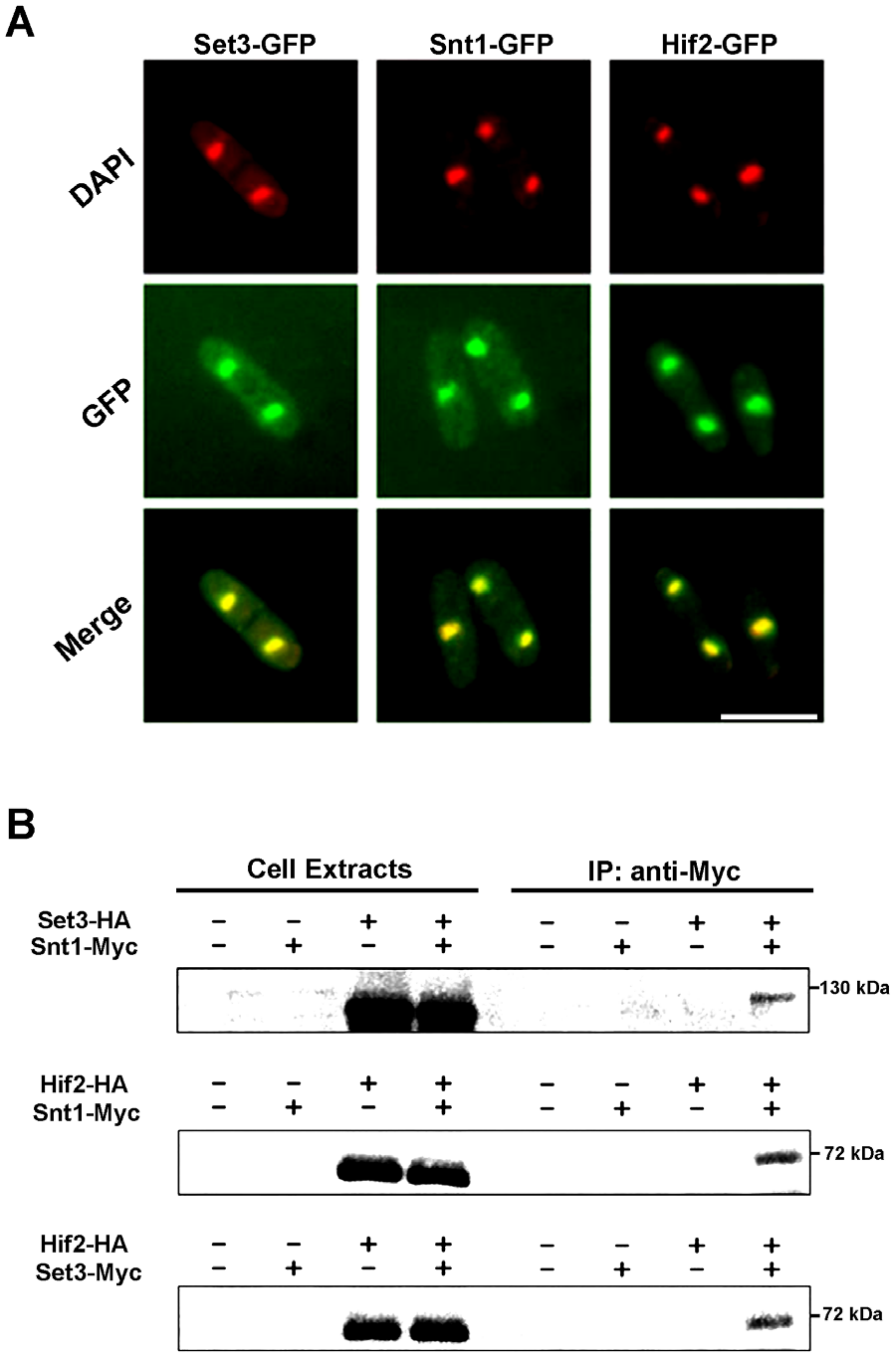
Hif2p, Set3p, and Snt1p form a nuclear-localized complex. (**A**) Cells expressing the indicated fusion proteins were grown to mid-log phase at 30°C in YES media, fixed, and then stained with DAPI and observed using the DAPI and GFP filter sets. Bar, 5 µm. (**B**) Cells expressing the indicated fusion proteins were grown to mid-log phase in YES, lysed under native conditions, and subjected to anti-Myc immunoprecipitations. Both total lysates and immunoprecipitates were resolved by SDS-PAGE and immunoblotted with antibodies specific for the HA epitope.

Bioinformatics suggested that the *hif2*, *set3*, and *snt2* gene products exist as part of a conserved histone deacetylase complex. In order to function in chromatin remodeling, one would expect Hif2p, Set3p, and Snt2p to localize to the nucleus. To test this prediction, strains expressing GFP-tagged fusion proteins – integrated at their normal genomic loci and under the control of their native promoters – were constructed. Consistent with a role in chromatin modification, all three proteins localized to the nucleus (Figure 4A). The localization of the fusion proteins was not altered as a function of cell cycle position or as a function of LatA concentration in the growth medium (data not shown).

Next, to determine if Hif2p, Set3p, and Snt1p physically interacted *in vivo*, co-immunoprecipitation experiments using Myc- or HA-epitope tagged alleles were performed. Interestingly, Set3-HA and Hif2-HA fusion proteins could be detected in anti-Myc immuno-precipitates of Snt1-Myc Set3-HA and Snt1-Myc Hif2-HA extracts (Figure 4B, top and middle panels). Likewise, Hif2-HA proteins could be detected in anti-Myc immuno-precipitates of Set3-Myc Hif2-HA extracts (Figure 4B, bottom panel). Taken together, these data demonstrate that Hif2p, Set3p, and Snt1p exist as part of a nuclear-localized protein complex in *S. pombe*.

### Protein levels of Set3p, Hif2p and Snt1p increase upon LatA treatment

**Figure 5:**
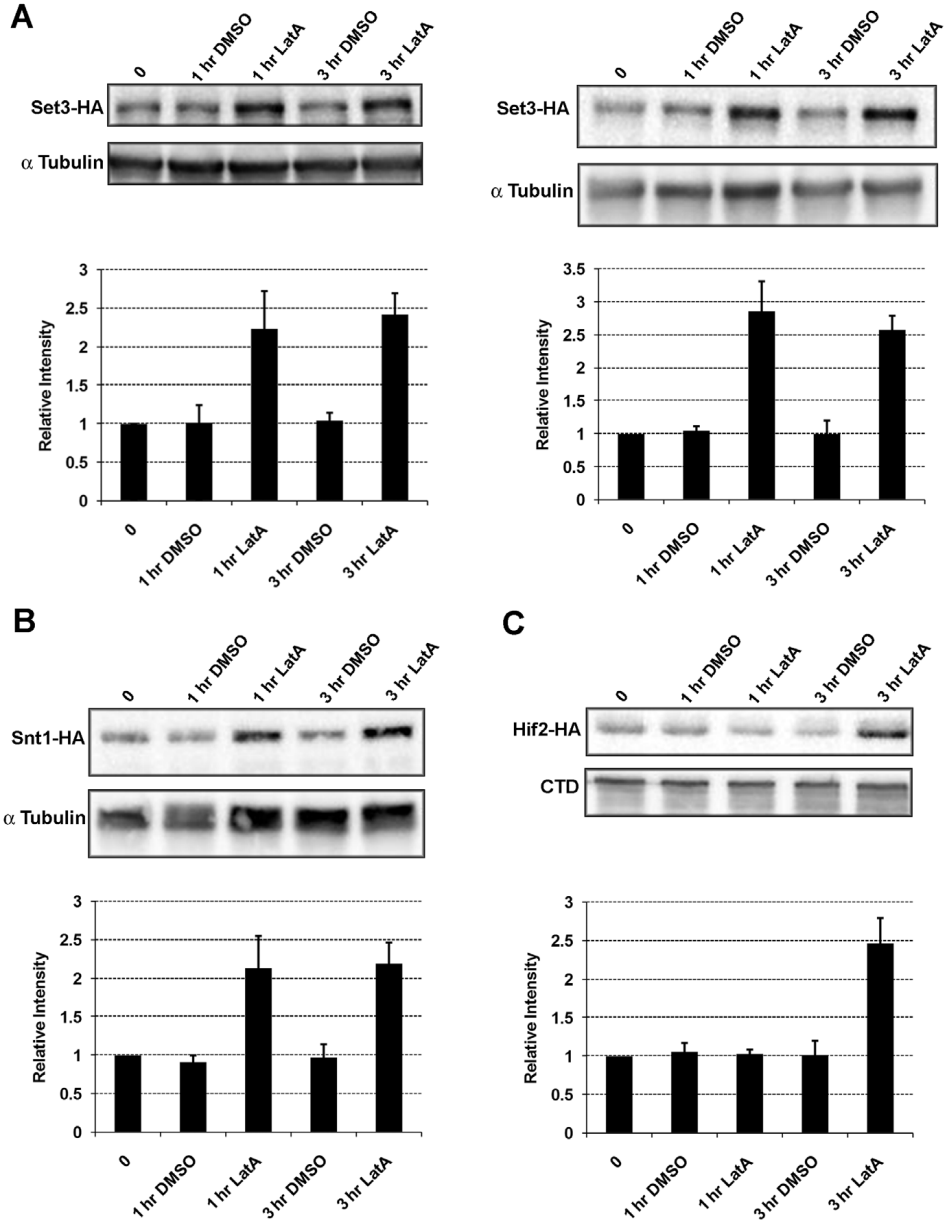
Set3p, Snt1p and Hif2p levels increase two- to three-fold upon treatment with low doses of LatA. (**A**) Strains expressing the indicated fusion proteins were grown to early log phase in YES at 30°C and treated with 0.5 µM LatA (left panel) or 1 µM LatA (right panel). Extracts were subjected to SDS-PAGE, transferred to PVDF membranes, and immunoblotted with anti-HA antibody. Tubulin was used as a loading control. (**B and C**) Strains expressing the indicated fusion proteins were grown to early log phase in YES at 30°C and treated with 0.5 µM LatA. Extracts were subjected to SDS-PAGE, transferred to PVDF membranes, and immunoblotted with anti-HA antibody. Tubulin or the RNA pol II carboxy-terminal domain (CTD) were used as loading controls. Signal intensities relative to the loading controls of three independent trials were quantified using ImageJ 1.41 Gel Analyzer software and are plotted below representative blots. Error bars indicate sd.

Since *hif2Δ*, *set3Δ*, and *snt2Δ* mutants display cytokinesis defects upon LatA treatment, we next asked if the expression levels of these proteins might be responsive to the presence of LatA in the growth medium. To this end, protein extracts were made from LatA (0.5 µM) or DMSO treated strains expressing Set3-HA, Snt1-HA and Hif2-HA fusion proteins. The levels of Set3-HA, Snt1-HA and Hif2-HA were then quantified by western blotting. Remarkably, while no significant changes were noted in DMSO treated cells, the protein levels of Set3p, Snt1p, and Hif2p increased 2–3 fold upon LatA treatment (Figure 5A–C). Increasing the dose of LatA to 1 µM did not increase the level of induction (compare Figure 5A, left and right panels). Thus, the magnitude of the increase is not dosage dependent in this range of LatA concentrations.

To ensure that changes in protein level were not due to genotype specific delays at a particular cell cycle stage (since wild-type cells engage the cytokinesis monitoring system, whereas the respective gene deletion mutants have this system abrogated) we examined the levels of the respective epitope tagged proteins upon release from a G2/M block produced through the use of the temperature sensitive *cdc25*-22 mutation (Figure S3A). The efficiency of the block and release was monitored by examining the level of bi-nucleate cells every 30 minutes after shifting from 36°C to 25°C (Figure S3B,C). The levels of Set3-HA, Snt1-HA, Hif2-HA were not significantly altered as a function of cell cycle position (Figure S3A). Taken together, these results are consistent with a model in which the increased activity of the Set3p-Snt1-Hif2p complex is required for a proper cellular response to LatA induced stress.

### Microarray expression profiling

Histone deacetylase complexes have well defined roles in regulating transcription through chromatin modification [23], [24]. This suggested a model in which the Set3p complex might affect the transcription of genes involved in regulating cytokinesis or the cytoskeleton. To further explore this hypothesis, expression profiles were generated for both wild-type and *set3Δ* mutants. Total RNA was extracted from wild-type or *set3Δ* cultures treated with LatA (0.5 µM) or DMSO for 3 hours. The RNA samples were then used in microarray hybridizations using Yeast Genome 2.0 Gene Chips purchased from Affymetrix. Three replicates of each strain under each growth condition (plus or minus LatA) were obtained for a total of 12 samples. After data processing (see Materials and Methods) the 12 samples were grouped according to two parameters: genotype (wild-type or *set3Δ*) and drug (LatA treated or DMSO treated). The complete data set, showing log_2_ normalized intensity values for all genes is included in File S1.

Next, to assess whether the expression data was providing meaningful biological results, the expression levels of genes known to play a role in the *S. pombe* core environmental stress response (CESR) were analyzed in wild-type cells [25]. The CESR defines a group of 240 genes whose expression is affected by a variety of different stresses. These stresses include extremes of temperature, osmolarity, salt concentration, as well as treatment with oxidizing or DNA damaging agents [25]. The expression of these genes is thought to define a transcriptional profile characterizing exposure to environmental stresses in *S. pombe*. Since LatA treatment would constitute an environmental stress, the expression of the CESR genes was analyzed in wild-type cells to determine if the microarray analysis could correctly detect changes in CESR gene expression.

**Figure 6:**
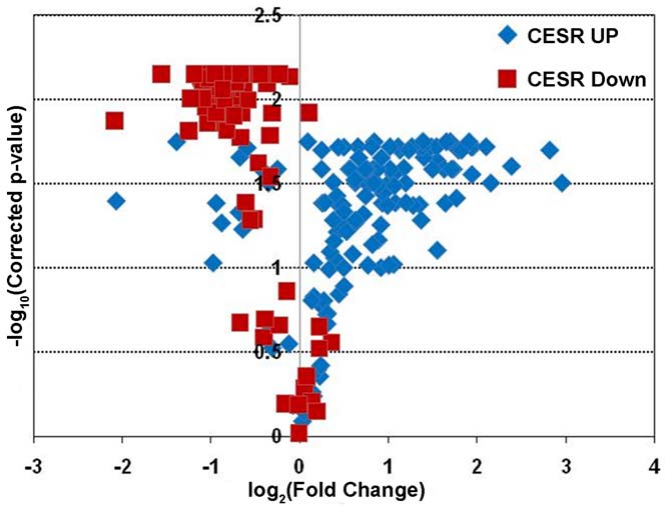
The core environmental stress response genes (CESR) respond to LatA treatment in wild-type cells. Volcano plot analysis of the expression of the CESR genes in wild-type strains treated with 0.5 µM LatA. Blue diamonds represent CESR genes normally up-regulated in response to multiple stresses. Red squares represent CESR genes normally down-regulated in response to multiple stresses.

The CESR is sub-divided into two groups: 136 genes that are up-regulated in response to stress (CESR-UP), and 104 that are down-regulated in response to stress (CESR-DN). When the expression of these genes was examined it was apparent that the majority of CESR-DN genes (96 out of 104) were down-regulated, whereas the majority of CESR-UP genes (120 out of 136) were up-regulated in LatA treated cultures relative to DMSO treated controls (Figure 6). Taken together these results indicated that the microarray analysis was providing accurate and biologically meaningful data.

**Figure 7:**
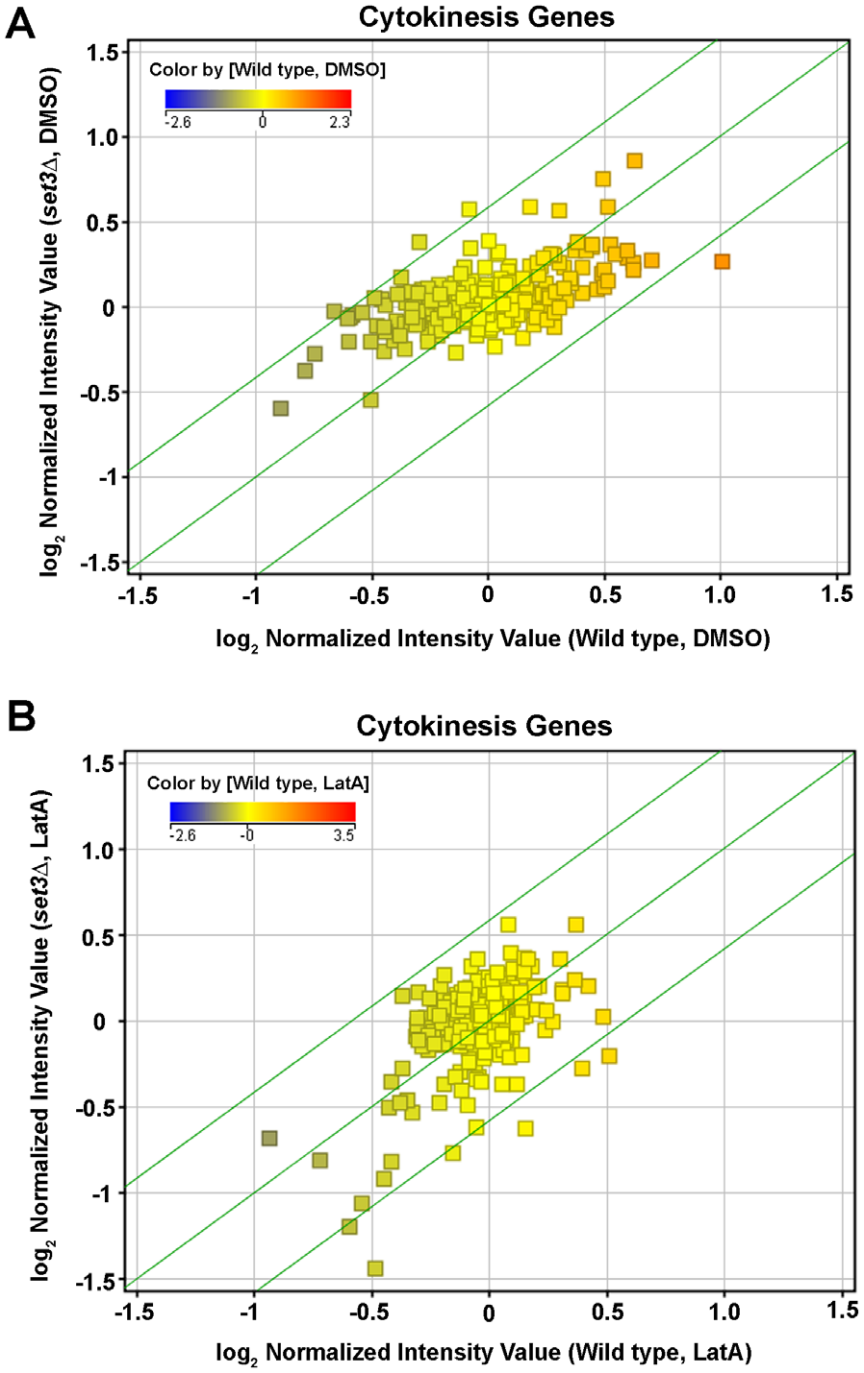
The expression levels of cytokinesis related genes are not differentially regulated. (**A**) Cells of the indicated genotypes were grown to mid-log phase at 30°C in YES, and then treated with DMSO (**A**) or 0.5 µM LatA (**B**) for 3 hours. Total RNA was extracted and used in expression profiling using Affymetrix Yeast 2.0. Genechips. Graphs shows scatter plot analysis comparing the expression of cytokinesis related genes in wild-type and *set3Δ* strains. Green lines represent the threshold for a 1.5 fold change in transcript levels. The color of the squares indicates the level of expression of that gene in DMSO (**A**) or LatA (**B**) treated wild-type cells.

For subsequent analysis, the data was grouped into four categories for comparison: 1) wild type, DMSO treated, 2) wild type, LatA treated, 3) *set3Δ*, DMSO treated and 4) *set3Δ*, LatA treated. In order to identify genes differentially regulated by genotype, volcano plots (p-value<0.05; fold change >1.5) were used to compare the gene expression profiles of wild-type and *set3Δ* mutant strains in both DMSO and LatA treated conditions. We first examined the expression of 333 genes with known roles in cytokinesis and/or the cytoskeleton (File S1 for a complete list). None of the genes were identified as being differentially expressed in response to LatA treatment. Furthermore, scatterplots comparing wild-type and *set3Δ* strains treated with either DMSO or LatA (Figure 7) showed a strong correspondence of expression for the vast majority of cytokinesis genes. Thus, the gene expression data did not support a model in which the Set3p complex plays a role in modulating the transcription of genes with roles in cytokinesis and/or the cytoskeleton.

**Figure 8:**
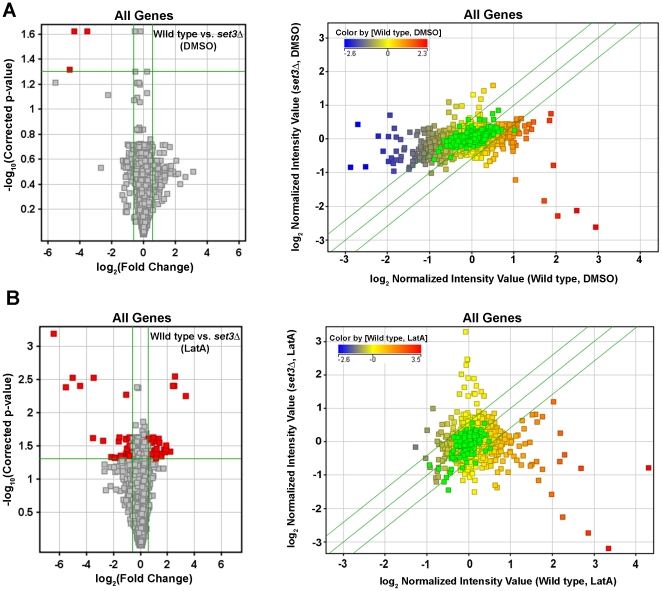
Identification of *S. pombe* genes differentially expressed with respect to genotype. Volcano plot (left panels) and scatter plot (right panels) analysis of the expression of all *S. pombe* genes in DMSO (**A**) or LatA (**B**) treated cells of the indicated genotype. Horizontal green lines in volcano plots represent a p-value of 0.05. Vertical green lines in volcano plots represent threshold for a 1.5 fold change in expression. Red squares in volcano plots indicate differentially expressed genes. Diagonal green lines in scatter plots represent the threshold for a 1.5 fold change in expression. The color of the squares in scatter plots indicates the level of expression of that gene in DMSO (**A**) or LatA (**B**) treated wild-type cells.

Since genes with roles in cytokinesis were not affected, we next examined the global data set to determine the genes that were differentially regulated by genotype. First, the expression profiles of *set3Δ* and wild-type strains were compared upon DMSO treatment. Surprisingly, only three genes were identified as being differentially regulated (p-value<0.05; fold change >1.5) (Figure 8A). As expected, this list included *set3* itself, as well as *mfm2*, and the uncharacterized ORF, SPAC186.05c. Thus, under normal growth conditions – where the *set3Δ* mutant shows no obvious phenotype – gene expression patterns were not altered to a great extent.

We next examined the difference in expression profiles in wild-type and *set3Δ* strains treated with LatA. Under these conditions 73 genes were identified as being differentially regulated (p-value<0.05; fold change >1.5) (Figure 8B). To determine if any of these genes shared any common functions, GO annotations were inspected. The gene ontology (GO) project is a bioinformatics initiative aimed at characterizing the attributes of genes and gene products across databases. This initiative describes all gene products with respect to their biological functions, molecular functions, and their cellular component (www.geneontology.org/). Interestingly, of the 73 differentially regulated genes, 40% (29 out of 73) were annotated by GO as having a role in the cellular response to stress (see File S1 for a complete list).

**Figure 9:**
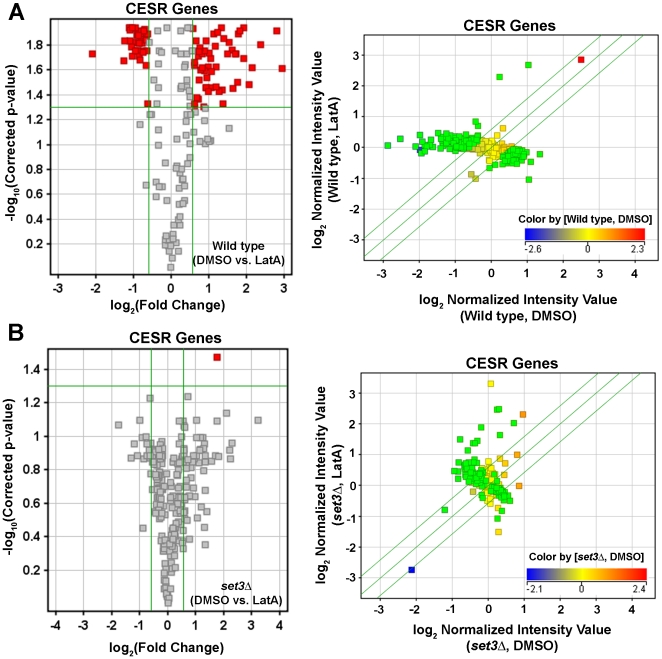
*set3Δ* mutants are impaired in their ability to modulate the expression of CESR genes in response to LatA treatment. Volcano plot (left panels) and scatter plot (right panels) analysis of the expression of CESR genes in wild-type (**A**) or *set3Δ* (**B**) strains in response to LatA treatment. Horizontal green lines in volcano plots represent a p-value of 0.05. Vertical green lines in volcano plots represent threshold for a 1.5 fold change in expression. Red squares in volcano plots indicate differentially expressed genes. Diagonal green lines in scatter plots represent the threshold for a 1.5 fold change in expression. The color of the squares in scatter plots indicates the level of expression of that gene in DMSO treated wild-type (**A**) or *set3Δ* (**B**) cells. Genes differentially expressed upon LatA treatment in wild-type cells are shown as green squares in scatter plots.

**Figure 10:**
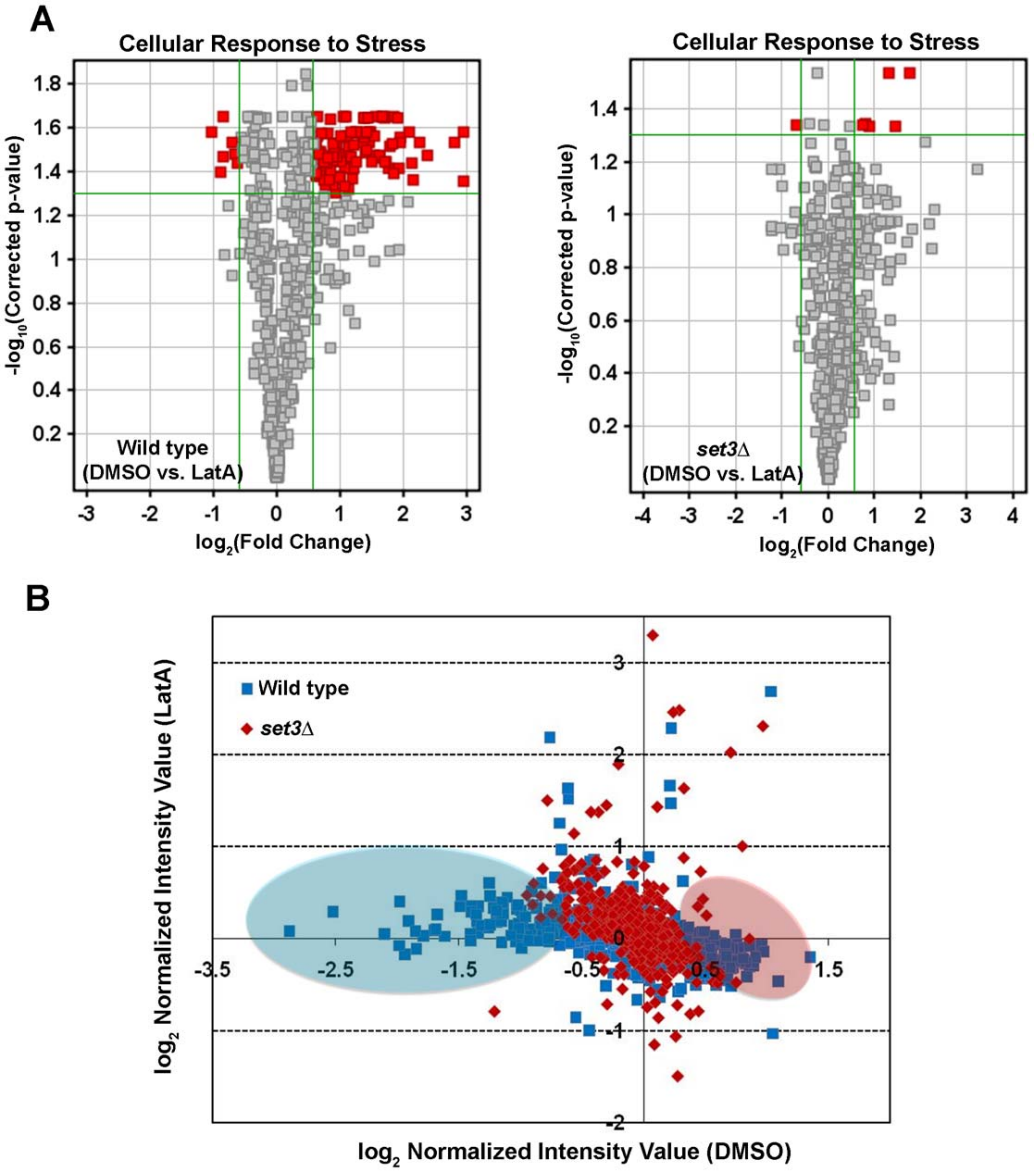
*set3Δ* mutants are impaired in their ability mount a proper transcriptional response to LatA treatment. (**A**) Volcano plot analysis of the expression of genes annotated by GO as having a cellular response to stress. The response of wild-type cells (left panel) and *set3Δ* mutants (right panel) treated with LatA are shown. Horizontal green lines represent a p-value of 0.05. Vertical green lines represent the threshold for a 1.5 fold change in expression. Red squares indicate differentially expressed genes. (**B**) Scatter plot analysis comparing the expression of stress response genes (both CESR genes and genes annotated by GO as having a cellular response to stress) in response to LatA treatment. Blue squares indicates data points for wild type cells. Red diamonds indicate data points for *set3Δ* mutants. Genes up-regulated in response to LatA in wild-type cells are highlighted with a blue oval. Genes down-regulated in response to LatA in wild-type cells are highlighted with a pink oval.

To further explore the role of Set3p in affecting stress response genes, we analyzed the expression of the CESR genes in LatA or DMSO treated wild-type cells. As expected 144 out of 240 genes (∼60%) were differentially expressed upon LatA treatment (Figure 9A). However, when the expression of CESR genes in LatA treated *set3Δ* cells was examined, only 1 out of 240 (<1%) were differentially regulated (Figure 9B). Next, we expanded the analysis to include any gene annotated by GO as having a cellular response to stress. In this sub-set, 113 out of 561 genes (∼20%) were differentially regulated upon LatA treatment in wild-type cells, while only 7 out of 561 genes (∼1%) were differentially regulated in *set3Δ* backgrounds (Figure 10A).

Lastly, we performed scatter plot analysis on a combined set of genes that included both the CESR and genes annotated by GO as having a cellular response to stress. Interestingly, scatter plot analysis of the stress genes in *set3Δ* mutants revealed that the distribution of data points was closely clustered around median levels (Figure 10B). In wild-type, on the other hand, a similar analysis of the distribution revealed the existence of genes that were either up- (Figure 10B, blue oval) or down-regulated (Figure 10B, pink oval) in response to LatA. Taken together these data indicate that *set3Δ* cells are severely impaired in their ability to mount a proper transcriptional response to LatA treatment.

## Discussion

*Schizosaccharomyces pombe* has proven to be an excellent model for understanding the regulatory modules that ensure the faithful and reliable execution of cytokinesis [1], [2], [3], [4], [6], [7], [8], [9], [10], [12], [13], [22]. In this report we further expand our knowledge in this area by identifying three components of a histone de-acetylase complex (Hif2p, Set3p, and Snt1p) whose function is required to ensure the successful completion of cytokinesis upon perturbation of the cell division machinery (Figures 1 and 2). Moreover, through the creation of *set3Δ lsk1Δ* and *set3Δ clp1Δ* double mutants, we demonstrate that the complex functions through a novel branch of control, independently of both Lsk1p and Clp1p (Figure 3). This is consistent with genetic data indicating that, unlike the *lsk1* gene deletion [13], *set3Δ* mutations are incapable of suppressing the lethal cytokinesis phenotype associated with SIN hyperactivation (data not shown). Lastly, through phenotypic analysis, co-immunoprecipitation data, and the analysis of intracellular localization, we provide support for a model in which Hif2p, Set3p, and Snt1p act together in a physical complex (Figures 2 and 4).

The importance of understanding the pathways required for the dependable execution of cytokinesis in eukaryotes was first articulated by Theodor Boveri almost 100 years ago [26]. In his classic work “*Concerning the origin of malignant tumours*” (1914) Boveri hypothesized that tetraploid intermediates – derived from either cytokinetic failure or cell fusion – might undergo chaotic multipolar mitoses leading to numerical and/or structural chromosomal defects. Recent experimental evidence provides strong support for Boveri's assertions. First, tetraploid mouse mammary epithelial cells generated by the inhibition of cytokinesis display increased rates of aneuploidy and (when transplanted into nude mice) give rise to malignant tumours at greater rates than controls [27]. Second, tetraploidy often precedes gross aneuploidy and is an early event in carcinogenesis [28], [29]. Third, aneuploidy (generated by loss of the kinesin, Kif4) promotes tumorigenesis in vivo [30]. Lastly, several tumor suppressors (BRCA2, LATS) are required for the completion of cytokinesis [31], [32]. Taken together these results suggest that mechanisms promoting the dependable execution of cytokinesis are important in maintaining genomic integrity and in preventing carcinogenesis [33], [34], [35]. Thus, in the broadest sense, an understanding of these pathways may provide a better understanding of one “route” by which eukaryotic cells become tumorigenic in multicellular organisms.

Given the importance of cytokinesis in maintaining genomic integrity it is particularly intriguing to note that orthologues of Hif2p, Set3p, and Snt1p exist in humans (*TBL1X*, *MLL5*, and *NCOR2*, respectively). Furthermore – as might be expected based on the selection criteria used in the genetic screen – MLL5, NCOR2, and TBL1X have themselves been shown to play a role in cytokinesis in human cells. In their study, Kittler et al. (2007) conducted a genome-wide RNAi screen aimed at identifying genes with roles in cell division in cultured HeLa cells. They discovered that the knockdown of *MLL5*, *TBL1X*, or *NCOR2* resulted in defects in furrow ingression, cytokinesis failure, and finally the generation of tetraploid intermediates with twice the normal number of centrosomes.

While highly speculative it is of interest to note that *MLL5* is found in a region of chromosome seven that is frequently deleted in myeloid malignancies, and furthermore that decreased *MLL5* expression levels correlate with unfavourable outcomes in patients with acute myeloid leukemia [36], [37]. Moreover, the down-regulation of the *NCOR2* gene can induce transformation in certain immortalized cell lines [38]. While a direct role in tumour progression via cytokinesis failure has not been shown, it is interesting to speculate as to whether *MLL5*, *NCOR2*, or *TBL1X* might indeed encode tumour suppressors, and if so, whether the loss of these genes, and any ensuing cytokinesis defects could be relevant to carcinogenesis. Regardless, the isolation of known human regulators of cytokinesis in this screen further supports the utility of using *S. pombe* as a model for the study of eukaryotic genetic regulatory networks.

A second observation of particular significance is the discovery that the levels of Set3p, Hif2p, and Snt1p increase 2–3 fold when wild-type cells are grown in the presence of low doses of LatA (Figure 5). Thus, in addition to the observed LatA hypersensitivity exhibited by the gene deletion mutants, these data provide further independent support that activity of the complex is required to respond properly to the presence of LatA in the growth medium. Up-regulation probably occurs at the post-transcriptional level since microarray data did not show strong induction of these genes in wild-type cells treated with LatA (File S1).

While the above data identifies the Set3p complex as being required for the proper response to LatA induced stress, it says little with respect to the mechanism of action. Since histone de-acetylase complexes have well defined roles in transcriptional regulation, we considered the possibility that the observed cytokinesis phenotypes were the result of defects in the transcription of genes involved in cytokinesis and/or the cytoskeleton (Figure 7). Importantly, while expression profiling clearly showed that this was not the case, a careful examination of the microarray data did reveal several interesting findings.

First, wild-type cells respond to LatA with a general cell stress response (Figures 6, 8,9,10). This was evident by the strong induction/repression of the fission yeast CESR genes. The CESR genes are predicted to modulate cellular metabolic pathways and to limit growth related processes [25]. It is hypothesized that activation of the CESR may promote survival against potentially lethal doses of a given stress and in doing so provide a means for the cell to adapt to its new environment [25], [39]. In stark contrast to wild-type cells, the *set3Δ* mutant exhibited a significantly reduced capacity to modulate the expression of stress response genes upon LatA treatment (Figures 8,9,10). Thus, cytokinetic failure in *set3Δ* mutants may be a manifestation of the mutant cells inability to properly adapt to the presence of LatA leading to direct and/or indirect effects on the function of the cytokinetic machinery. It is also of interest to note that, in addition to LatA, *set3Δ* strains show sensitivity to the calcineurin inhibitor, FK506 [40]. Intriguingly, calcineurin mutants in *S. pombe* have been shown to affect cytokinesis, cell polarity, and spindle pole body positioning [41].

A role in the stress response, may be an evolutionarily conserved feature since the Set3p complex in budding yeast is required to respond to secretory stress [42]. Furthermore, the budding yeast class I histone deacetylase Rpd3p, and its associated Rpd3-L complex, is required for activation and repression of environmental stress response genes [43]. Taking this into consideration, we favour a model in which the observed defects in cytokinesis are related to the impaired ability of the mutants to modulate gene expression so as to properly counter the effects of LatA induced stress. This is supported by the observation that the protein levels of all three complex members increase in response to LatA (Figure 5), as well as the observation that wild-type cells modulate the expression of a large sub-set of genes with a role in the stress response (Figures 6, 9 and 10). In any event, we suspect that future analysis of this system might translate into a theoretical framework for understanding how the orthologous MLL5 complex functions to regulate cytokinesis in human cells, as well as how its dysfunction might lead to genomic instability.

## Materials and Methods

### Yeast Methods

All *Schizosaccharomyces pombe* strains used in this work (Table S1) originated from previous studies [12], [44], [45], were created during the course of this work, or were purchased from Bioneer Corporation (Alameda, CA). *Schizosaccharomyces pombe* cells were cultured in either YES or Edinburgh Minimal Media (EMM) (Forsburg and Rhind 2006) with the appropriate supplements (Adenine, Histidine, Leucine, or Uracil). Liquid cultures were grown with shaking (200 rpm) at 30°C. Genetic crosses were performed using standard methods [46]. In experiments involving Latrunculin treatment, *S. pombe* cells were grown to mid log phase (O.D. 0.2) and treated with 0.2–0.5 µM of Latrunculin A (Enzo Life Sciences International, Plymouth Meeting, Pennsylvania) dissolved in DMSO. Cells were grown at 30°C with shaking at 200 rpm for 3–6 hrs, before being fixed. All experiments were repeated a minimum of three times. Plasmid vectors were transformed into *S. pombe* using the lithium acetate protocol according to Forsburg and Rhind [46]. In block and release experiments, *cdc25-22* cells were grown to logarithmic phase in YES at 25°C, shifted to 36°C for 3 hours to block at the G2/M transition, and then shifted back to 25°C to effect release of the block. The cell cycle progress of the strains was subsequently monitored by determining the level of bi-nucleate cells every 30 minutes after the shift.

### Fluorescence Microscopy

*S. pombe* cells expressing Lsg1-GFP fusions, were fixed using ethanol fixation [46] and stored in PBS pH 7.4. To observe nuclei and cell wall/septa material, cells were mixed with 0.02 µg/µL 4′6,-diamidino-2-phenylindole (DAPI) and 1 µg/µL aniline blue. Fluorescent images were obtained using Zeiss Axioskop 2 microscope driven by ImageJ 1.41 software (National Institutes of Health) and Scion CFW Monochrome CCD Firewire Camera (Scion Corporation, Frederick Maryland) using DAPI and GFP filter sets.

### Cloning Methods

The *snt1* gene deletion mutant was created using a PCR based cloning strategy. A 286 bp region upstream of the *snt1* start codon was PCR amplified using High-Fidelity PCR Enzyme Mix (Fermentas Life Sciences) with the forward primer 5′-ggg ggg gta cca aat gaa ggg gat tcc ttg g-3′ and reverse primer 5′-ggg ggc tcg agt gtc aga gga ggc act aca gc-3′ and cloned into the pSKURA4 vector upstream of the *ura4* selectable marker using the restriction enzymes *Kpn*I and *Xho*I (Fermentas Life Sciences). Next, a 259 bp region downstream of the *snt1* stop codon was PCR amplified using the forward primer 5′-ggg ggt cta gat gtg tcg ggt tat gat ggt g-3′ and reverse primer 5′-ggg ggg agc tca ttt ttg gtg tcg gtt ttg c-3′ and cloned downstream of the *ura4* selectable marker in pSKURA4 using the restriction enzymes *Xba*I and *Sac*I. Molecular cloning of the desired fragments was confirmed by restriction digestion and DNA sequencing. The *ura4* selectable marker flanked by upstream and downstream regions of *snt1* was excised with restriction enzymes *Kpn*I and *Sac*I to isolate a linear dsDNA deletion cassette. The deletion cassette was transformed into *S. pombe* strain MBY1343 (*ura4-D18*). Ura4^+^ integrants were selected for by growth on EMM lacking uracil and subjected to colony PCR to identify clones in which the construct had integrated into the genome via homologous recombination. Strains bearing gene deletions of *set3* and *hif2* were purchased from Bioneer Corporation (Alameda, CA). Genotypes were verified by colony PCR. The *hif2::natMX* gene deletion was created using the high throughput knockout strategy devised by the Kim Nasmyth lab [47]. Primers, plasmids and a detailed protocol are available at the *S. pombe* deletion web server (http://mendel.imp.ac.at/Pombe_deletion/).

*S. pombe* strains expressing carboxy-terminal epitope tagged fusion protein were constructed using a PCR based cloning strategy. To create the Set3-GFP and Set3-HA expressing strains a C-terminal fragment of the *set3* gene was PCR amplified using High-Fidelity PCR Enzyme Mix (Fermentas Life Sciences) from *S. pombe* genomic DNA with the forward primer 5′-ggg ggg aat tct gaa ata ctt caa gaa gcg aaa aca ag-3′ and reverse primer 5′-ggg ggc ccg ggt cgc gta aat gaa ggg tta g-3′ and cloned in frame into the *EcoRI* and *SmaI* sites of the pJK210-GFP and pJK210-HA vectors respectively. Molecular cloning of the desired C-terminal fragments was confirmed by restriction digestion and DNA sequencing. Plasmid clones containing the desired C-terminal fragment were transformed into *S. pombe* strain MBY1343 (*ura4-D18*). Ura4^+^ integrants were selected for by growth on EMM lacking uracil and subjected to colony PCR to identify clones in which the construct had integrated into the genome via homologous recombination. To create the Set3-myc expressing strain a C-terminal fragment of the *set3* gene was PCR amplified using High-Fidelity PCR Enzyme Mix (Fermentas Life Sciences) from *S. pombe* genomic DNA with the forward primer 5′-ggg ggg gta cct gaa ata ctt caa gaa gcg aaa aca ag-3′ and reverse primer 5′- ggg ggc ccg ggt cgc gta aat gaa ggg tta g-3′ and cloned in frame into the *KpnI* and *SmaI* sites of the pJK210-Myc vector. Plasmid clones containing the desired C-terminal fragment were transformed into *S. pombe* strain MBY1343 (*ura4-D18*). Ura4^+^ integrants were selected for by growth on EMM lacking uracil and subjected to colony PCR to identify clones in which the construct had integrated into the genome via homologous recombination.

Snt1-GFP and Snt1-Myc as well as Hif2-GFP and Hif2-HA expressing strains were created using an analogous strategy as the epitope tagged Set3p strains. The forward and reverse primers used to create the Snt1-GFP integrant strain were 5′-ggg ggg aat tct gag gtt ggg atg aaa aag aag aa-3′ and 5′-ggg ggc ccg ggt aca att tta tcg ttt ttg gac tg-3′ respectively, and the forward and reverse primers used to create the Snt1-Myc integrant strain are 5′-ggg ggg gta cct gag gtt ggg atg aaa aag aag aa-3′ and 5′-ggg ggc ccg ggt aca att tta tcg ttt ttg gac tg-3′ respectively. The forward and reverse primers used to create the Hif2-GFP and Sif3-HA integrant strains are 5-ggg ggg aat tct gat cta gag gtg atg ctg gtg c-3′ and 5′-ggg ggc ccg ggc aga gaa tca tgt aaa aaa tca ca-3′ respectively.

### Biochemical and Immunological Methods

Cells of the indicated genotype were grown up to the mid-log phase at 30°C, collected by centrifugation, and resuspended in STOP buffer (10 mM Tris-HCl pH 8.0, 150 mM NaCl, 50 mM NaF, 10 mM EDTA, 1 mM NaN_3_). Cell pellets were stored at −80°C up to a maximum of 6 months. Cell pellets were subsequently thawed, and lysed using vortexing with glass beads in extraction buffer (1% IGEPAL CA630 (tetr-Octylphenoxy polyethanol), 150 mM NaCl, 50 mM Tris-HCl pH 8.0, 2 mM EDTA, 1 mM PMSF (phenylmethanesulphonylfluoride), 2 mM Benzamidine, 50 mM NaF, 0.1 mM Na_3_VO_4_, 50 mM B-glycerophosphate, 15 mM p-nitrophenyl phosphate, ¼ Tablet Sigma Protease Inhibitors). Total cell extracts were subjected to SDS-PAGE, transferred to PVDF membranes and immunoblotted with anti-HA primary antibody (HA.11; Sigma) at a dilution of 1∶2000. As a loading control, anti-tubulin (B14; Sigma) was used at a dilution of 1∶2000, or primary antibody specific to the unphosphorylated C-terminal domain of RNA polymerase II (8WG16; Covance) was used at a dilution of 1∶5000. Peroxidase conjugated anti-mouse IgG (Sigma) at 1∶10000, was used as secondary antibody for HA, tubulin and RNA polymerase II blotted membranes. Blots were quantified using ImageJ 1.41 gel analyzer software (National Institutes of Health).

In co-immunoprecipitation experiments cell extracts were obtained from the indicated strains as described above. Immunoprecipitations were performed using Protein G Dynabeads® (Invitrogen) according to the manufacturer's protocol. Briefly, immunoprecipitations were performed by incubating anti-HA antibodies (HA.11; Sigma) with the Protein G Dynabeads using extraction buffer as the wash solution. Cell extracts were then added to the antibody-bound bead slurry. After incubation and repeated washing with extraction buffer, the bound proteins were eluted by incubation at 96°C for 5 minutes. The eluted proteins were then subjected to SDS-PAGE, transferred to PVDF membranes and probed with anti-Myc antibodies (9E10; Sigma).

### Global Gene Expression Analysis

Strains of the indicated genotype were grown to mid-log phase and treated with 0.5 µM LatA or an equal volume of DMSO for 3 hours at 30°C. Total RNA extraction was performed using the Ambion RiboPure™ Yeast Kit (Ambion Inc). Three replicates were performed for each strain under each growth condition (plus or minus LatA). Bioanalysis of all samples showed that the isolated RNA was of sufficient quality to proceed to hybridizations (data not shown). GeneChip® Yeast Genome 2.0 Arrays purchased from Affymetrix were used for hybridization. Hybridizations were performed by the London Regional Genomic Centre using standard Affymetrix protocols. The quality of hybridization was analyzed using duplicate probes for the *bioB*, *bioC*, *bioD*, and *cre* genes. During hybridization, transcripts of these genes are “spiked” into the Affymetrix hybridization cocktail at concentrations of 1.5, 5, 25 and 100 pM, respectively. The level of hybridization as measured by the normalized signal values (data not shown) was consistent with the level of spiked transcript (i.e. *bioB* showed the lowest signal values and *cre* the highest). This demonstrated that the hybridizations had been performed successfully and that the data could be used for further analysis.

Expression profiling data was obtained from London Regional Genomic Centre as .CEL files, which contained the raw hybridization signal-intensity values. Analysis of the data was done using Genespring GX 10.0.1 software provided by Agilent Technologies Inc. and Strand Life Sciences Pvt. Ltd. The .CEL files were first normalized with RMA (robust multi-array analysis) algorithm. RMA uses PM (perfect match) probes from the data and corrects the background by fitting a model that is the addition of an exponentially distributed signal and a normally dispersed background [48]. This generated normalized hybridization signal-intensity data for all twelve samples. Normalized expression data was subsequently used in the analysis. Data was grouped into two categories: genotype (wild type or *set3Δ*), and b) drug (LatA or DMSO treated). Thus, four experimental groups were used for comparison: 1) wild type, DMSO treated, 2) wild type, LatA treated, 3) *set3Δ*, DMSO treated, and 4) *set3Δ*, LatA treated. To identify differentially regulated genes, data was filtered using Volcano plot analysis employing the Benjamin-Hochberg multiple testing correction. Genes showing statistically significant differences (p-value<0.05) and fold changes greater than 1.5 were identified as differentially regulated. Data is accessible from the GEO database under accession number GSE33228).

## Supporting Information

Figure S1 **ClustalW alignments of the Hif2p, Set3p, and Snt1p proteins with their human orthologues, TBL1X (A), MLL5 (B), and NCOR2 (C), respectively.** Alignments in (**B**) and (**C**) were performed with conserved segments present in the N-terminal regions of the respective proteins. Asterisks (*) indicate identities. Colons (**:**) indicate conserved substitutions. Periods (**.**) indicate semi-conserved substitutions.
(TIF)

Figure S2 **Deletion of the** ***set3*** **,** ***snt1*** **, or** ***hif2*** **genes reduces the restrictive temperature of** ***cdc15-140*** **mutants.** Cells of the indicated genotype were cultured to logarithmic growth phase at 25°C. Ten-fold serial dilutions were subsequently plated onto solid YES media and incubated at 25°C, 31°C, or 36°C for 3 d.
(TIF)

Figure S3 **The protein levels of Set3-HA, Snt1-HA, or Hif2-HA do not change as a function of cell cycle position.** Strains of the indicated genotype were grown to early log phase in YES at 25°C and shifted to 36°C for 3 hours to arrest the cells at the G2/M transition. Cells were subsequently released from the block by shifting to 25°C and cells collected every 30 minutes. (**A**) Extracts were subjected to SDS-PAGE, transferred to PVDF membranes, and immunoblotted with anti-HA antibody. Tubulin was used as a loading control. (**B**) To monitor the efficiency of the block and release, the level of bi-nucleate cells was quantitated every 30 minutes after shift to 25°C. (**C**) Representative micrographs of Set3-HA *cdc25-22* cells at various time points after release. Cells were fixed and stained with DAPI (nuclei) and aniline blue (cell wall/septa).
(TIF)

Table S1 **Yeast strains used in this study.**
(DOC)

File S1 (XLSX)
