# Supplementary figures and images for: The SET Domain Protein, Set3p, Promotes the Reliable Execution of Cytokinesis in Schizosaccharomyces pombe
Source: PLoS One. 2012 Feb 8;7(2):e31224. doi: 10.1371/journal.pone.0031224 (PMC3275627; doi:10.1371/journal.pone.0031224)

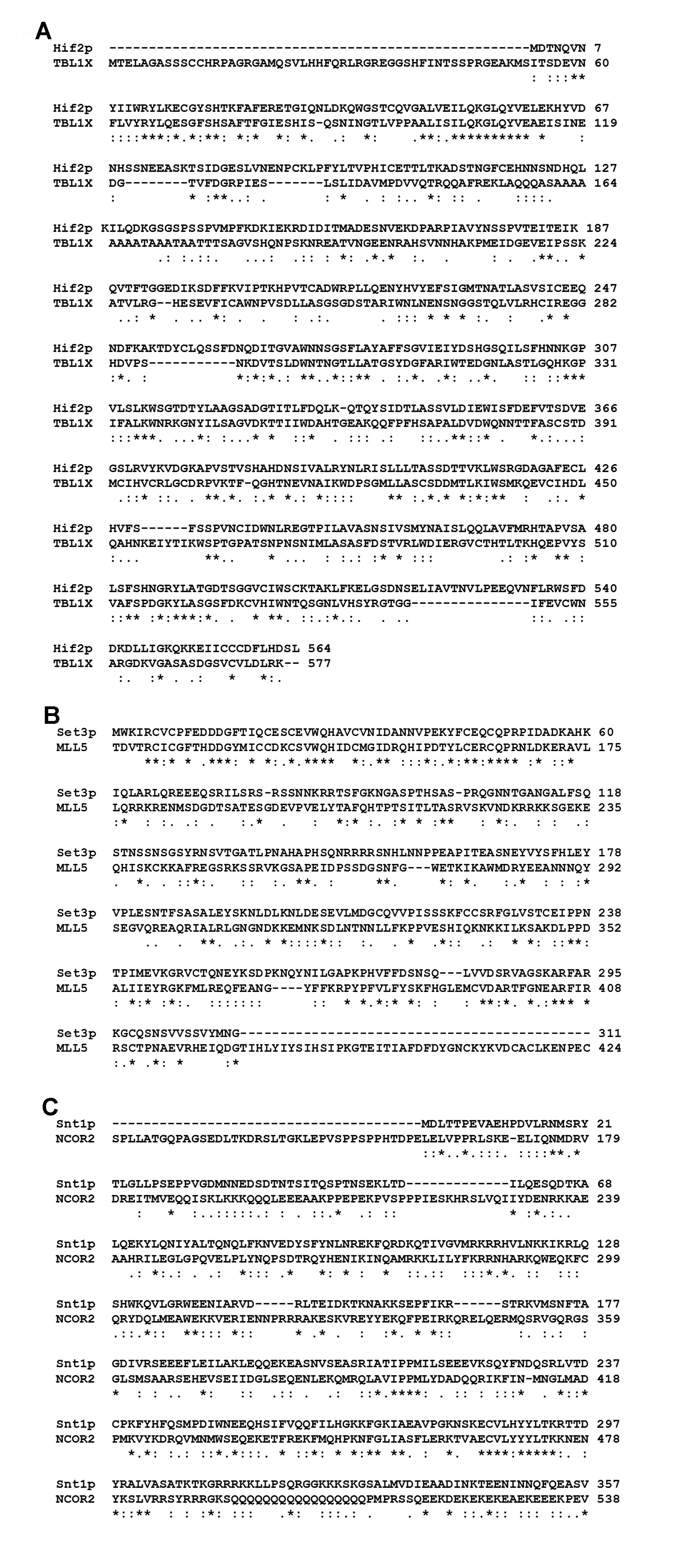

Supplement: Figure S1 — ClustalW alignments of the Hif2p, Set3p, and Snt1p proteins with their human orthologues, TBL1X (A), MLL5 (B), and NCOR2 (C), respectively. Alignments in (B) and (C) were performed with conserved segments present in the N-terminal regions of the respective proteins. Asterisks (*) indicate identities. Colons (:) indicate conserved substitutions. Periods (.) indicate semi-conserved substitutions. (TIF) [file pone.0031224.s001.tif]

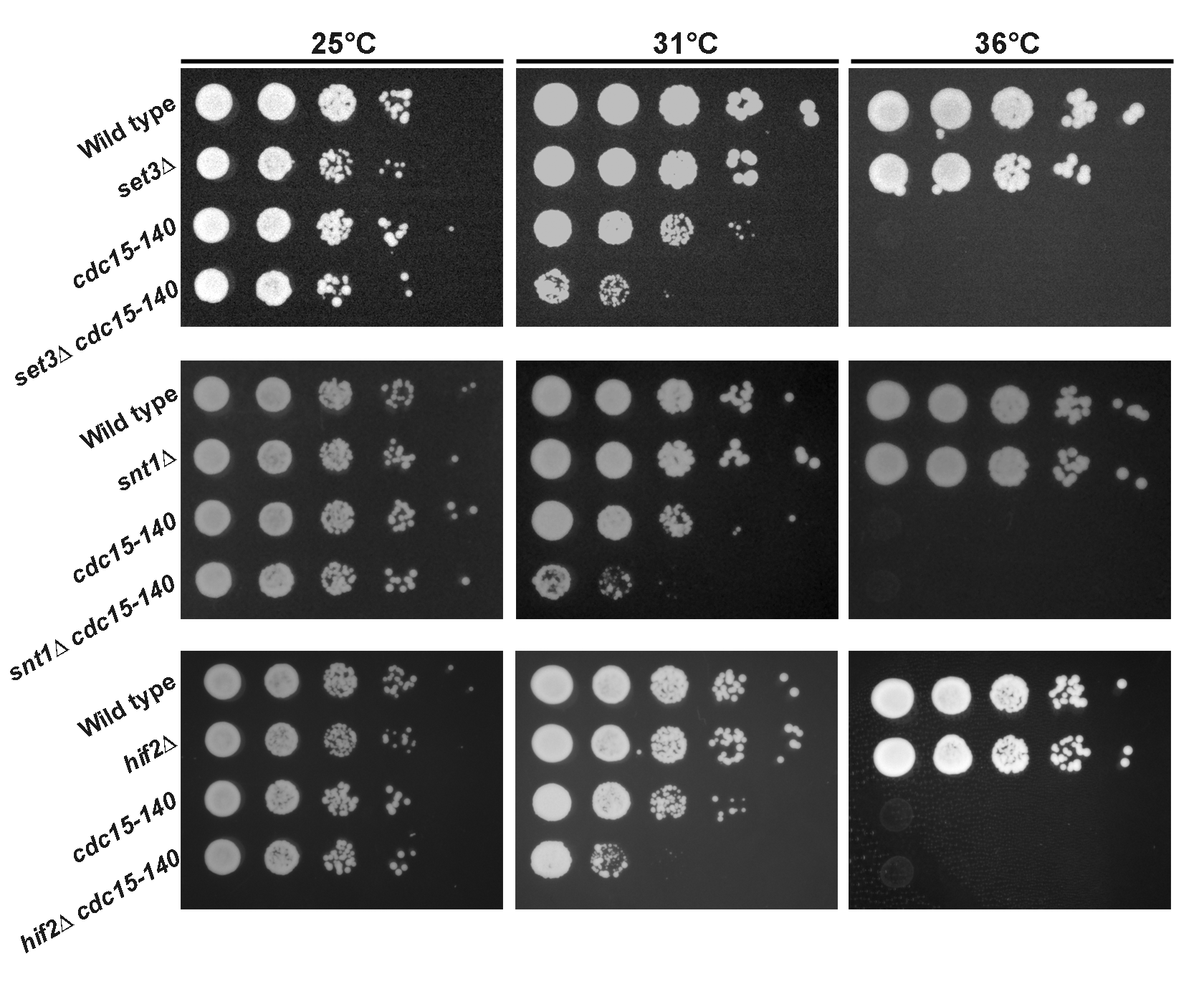

Supplement: Figure S2 — Deletion of the set3 , snt1 , or hif2 genes reduces the restrictive temperature of cdc15-140 mutants. Cells of the indicated genotype were cultured to logarithmic growth phase at 25°C. Ten-fold serial dilutions were subsequently plated onto solid YES media and incubated at 25°C, 31°C, or 36°C for 3 d. (TIF) [file pone.0031224.s002.tif]

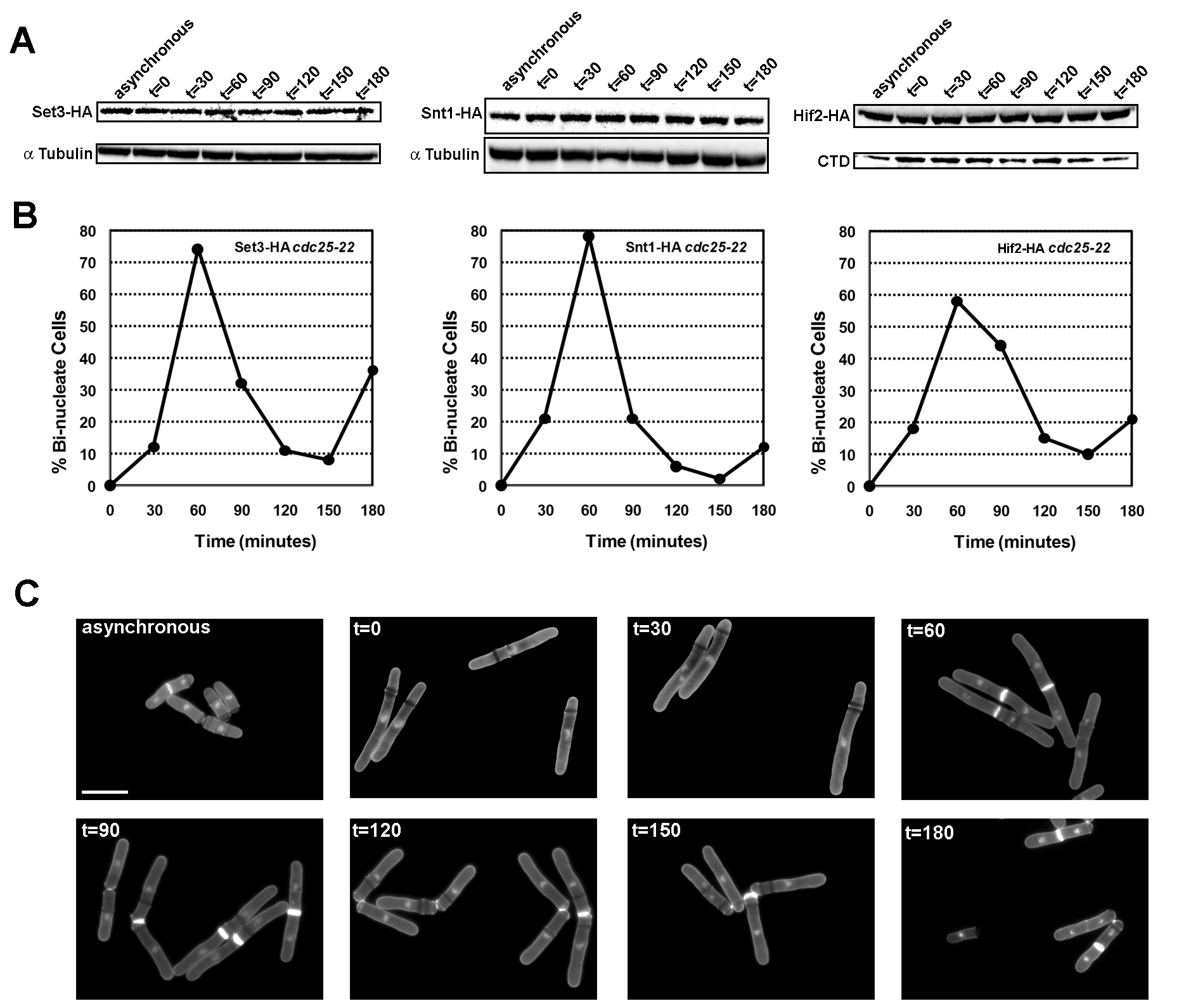

Supplement: Figure S3 — The protein levels of Set3-HA, Snt1-HA, or Hif2-HA do not change as a function of cell cycle position. Strains of the indicated genotype were grown to early log phase in YES at 25°C and shifted to 36°C for 3 hours to arrest the cells at the G2/M transition. Cells were subsequently released from the block by shifting to 25°C and cells collected every 30 minutes. (A) Extracts were subjected to SDS-PAGE, transferred to PVDF membranes, and immunoblotted with anti-HA antibody. Tubulin was used as a loading control. (B) To monitor the efficiency of the block and release, the level of bi-nucleate cells was quantitated every 30 minutes after shift to 25°C. (C) Representative micrographs of Set3-HA cdc25-22 cells at various time points after release. Cells were fixed and stained with DAPI (nuclei) and aniline blue (cell wall/septa). (TIF) [file pone.0031224.s003.tif]
