# Supplementary material for: The SET Domain Protein, Set3p, Promotes the Reliable Execution of Cytokinesis in Schizosaccharomyces pombe
Source: PLoS One. 2012 Feb 8;7(2):e31224. doi: 10.1371/journal.pone.0031224 (PMC3275627; doi:10.1371/journal.pone.0031224)
Supplement: Table S1 — Yeast strains used in this study. (DOC) [file pone.0031224.s004.doc]

| **Table S1: Yeast strains used in this study.** | | |
| --- | --- | --- |
| **Strain Name** | **Relevant Genotype** | **Source** |
| **SR2** | *clp1::ura4^+^ set3::KanMX ura4-D18* | This Study |
| **SR3** | *lsk1::ura4^+^ set3::KanMX ura4-D18* | This Study |
| **SR4** | *set3-GFP::ura4^+^ ura4-D18 h^+^* | This Study |
| **SR5** | *set3-HA::ura4^+^ ura4-D18 h^+^* | This Study |
| **SR10** | *hif2-HA::ura4^+^ ura4-D18 h^+^* | This Study |
| **SR11** | *hif2-myc::ura4^+^ ura4-D18 h^+^* | This Study |
| **SR14** | *snt1-GFP::ura4^+^ ura4-D18 h^+^* | This Study |
| **SR15** | *snt1-HA::ura4^+^ ura4-D18 h^+^* | This Study |
| **SR16** | *snt1-myc::ura4^+^ ura4-D18 h^+^* | This Study |
| **SR20** | *set3::KanMX ura4-D18 leu‐32 ade6‐216 h^+^* | This Study |
| **SR22** | *hif2::KanMX ura4-D18 leu‐32 ade6‐216 h^+^* | This Study |
| **SR35** | *set3-HA::ura4^+^ snt1-myc::ura4^+^ ura4-D18* | This Study |
| **SR36** | *set3-myc::ura4^+^ hif2-HA::ura4^+^ ura4-D18* | This Study |
| **SR37** | *snt1-myc::ura4^+^ hif2-HA::ura4^+^ ura4-D18* | This Study |
| **SR40** | *hif2-GFP::ura4^+^ ura4-D18 h^+^* | This Study |
| **SR45** | *snt1::ura4^+^ ura4-D18 h^+^* | This Study |
| **RS47** | *ura4‐D18 leu‐32 ade6‐216 h^‐^* | JK Collection |
| **MBY154** | *cdc15-140 ade6-21x leu1-32 h^-^* | JK Collection |
| **MBY1343** | *ura4‐D18 leu^+^ h^+^* | JK Collection |
| **JK9** | *clp1::ura4^+^ ura4-D18 h^-^* | JK Collection |
| **JK29** | *lsk1::ura4^+^ ura4-D18 leu1-32 h^-^* | JK Collection |
| **JK697** | *set3::KanMX cdc15-140 ade6-21x leu1-32* | This Study |
| **JK699** | *set3-HA::ura4^+^ cdc25-22 ura4-D18* | This Study |
| **JK700** | *snt1-HA::ura4^+^ cdc25-22 ura4-D18* | This Study |
| **JK701** | *hif2-HA::ura4^+^ cdc25-22 ura4-D18* | This Study |
| **JK715** | *snt1::ura4 cdc15-140 ura4-D18* | This Study |
| **JK730** | *hif2::NatMX ura4-D18 leu1-32 ade6-216 his3-D1h^-^* | This Study |
| **JK731** | *set3::KanMX hif2::NatMX ura4-D18 leu‐32 ade6‐216 h^+^* | This Study |
| **JK732** | *snt1::ura4^+^ hif2::NatMX ura4-D18 h^+^* | This Study |
| **JK733** | *set3::KanMX snt1::ura4 ura4-D18* | This Study |
| **JK734** | *set3::KanMX snt1::ura4 hif2::NatMX ura4-D18* | This Study |
| **JK736** | *hif2::NatMX cdc15-140 ade6-21x leu1-32* | This Study |
